# Magnetic Properties and Microstructure of FeCoNi(CuAl)_0.8_Sn_*x*_ (0 ≤ *x* ≤ 0.10) High-Entropy Alloys

**DOI:** 10.3390/e20110872

**Published:** 2018-11-13

**Authors:** Zhong Li, Chenxu Wang, Linye Yu, Yong Gu, Minxiang Pan, Xiaohua Tan, Hui Xu

**Affiliations:** 1 Institute of Materials Science, School of Materials Science and Engineering, Shanghai University, Shanghai 200072, China; 2 Shanghai Marine Diesel Engine Reserch Institute, Shanghai 201108, China; 3 Qianjiang College, Hangzhou Normal University, Hangzhou 310036, China

**Keywords:** high-entropy alloys (HEAs), phase constitution, magnetic properties, Curie temperature, phase transition

## Abstract

The present work exhibits the effects of Sn addition on the magnetic properties and microstructure of FeCoNi(CuAl)_0.8_Sn*_x_* (0 ≤ *x* ≤ 0.10) high-entropy alloys (HEAs). The results show all the samples consist of a mixed structure of face-centered-cubic (FCC) phase and body-centered-cubic (BCC) phase. The addition of Sn promotes the formation of BCC phase, and it also affects the shape of Cu-rich nano-precipitates in BCC matrix. It also shows that the Curie temperatures (*T_c_*) of the FCC phase and the saturation magnetization (*M_s_*) of the FeCoNi(CuAl)_0.8_Sn*_x_* (0 ≤ *x* ≤ 0.10) HEAs increase greatly while the remanence (*B_r_*) decreases after the addition of Sn into FeCoNi(CuAl)_0.8_ HEA. The thermomagnetic curves indicate that the phases of the FeCoNi(CuAl)_0.8_Sn*_x_* (0 ≤ *x* ≤ 0.10) HEAs will transform from FCC with low *T_c_* to BCC phase with high *T_c_* at temperature of 600–700 K. This work provides a new idea for FeCoNi(CuAl)_0.8_Sn*_x_* (0 ≤ *x* ≤ 0.10) HEAs for their potential application as soft magnets to be used at high temperatures.

## 1. Introduction

Since the first report of high-entropy alloys (HEAs) in 2004 [1], researchers have shown an increased interest in the study of HEAs. HEAs are the definition of alloys that are typically composed of more than 5 principal elements, which have broken the traditional alloy design concept based on 1 or 2 principal elements [2]. In contrast with the conventional alloys, HEAs predominantly trend to form an amorphous structure [3,4] or a simple solid solution with body-centered-cubic (BCC) phase [5,6], face-centered-cubic (FCC) phase [7,8,9] or a mixture of them [10,11], which is attributed to the high mixing entropy of HEAs [12,13]. The unique design concept and the significant mixing entropy effect of HEAs give them potential application in many high-entropy structural and functional materials. For example, HEAs have huge potential for use in jet-engine turbines, thin-film resistors, heat- or wear-resistant parts, functional coatings, and electronic products [13,14]. Recently, the good magnetic properties of HEAs capture the increasing interest in this new field of materials [15,16]. It is worth noting that many HEAs [17,18,19,20] consist of several ferromagnetic elements, such as Fe, Co, and Ni, and they also have a good comprehensive mechanical properties, which make them have great application potential in soft magnetic materials. HEAs with good magnetic and mechanical properties are expected to be used in electric motors, electromagnets, and magnetic recording. Liu et al. [21,22] reported the FeCoNi_0.25_Al_0.25_ HEA exhibits a high saturation magnetization (*M_s_* = 101.0 emu/g) and a low coercivity (*H_c_* = 268 A/m). Zuo et al. [23] found CoNiMnGa HEA shows a low saturation magnetostriction coefficient and a high Curie temperature (*T_c_*). In our previous work [24], the FeCoNi(CuAl)_0.8_ HEA consisting of BCC and FCC phases shows good magnetic and mechanical properties, and it was found that BCC phases show a higher *M_s_* than that of FCC phases for the FeCoNi(CuAl)_0.8_ HEA. The other work of our group [25] found a minor amount of Ga addition into FeCoNi(CuAl)_0.8_ HEA can promote the formation of BCC phase and improve the *M_s_* of the alloy, and the value of remanence (*B_r_*) and coercivity (*H_c_*) also increases. It was reported that the addition of Sn can hinder the formation of FCC phase [26] and promote the formation of BCC phase [27]. Therefore, in this work, a minor amount of Sn was added into the FeCoNi(CuAl)_0.8_ HEA hoping to get a higher volume fraction of BCC phase.

In this work, the FeCoNi(CuAl)_0.8_Sn*_x_* (0 ≤ *x* ≤ 0.10) HEAs were studied from phase constitutions to microstructure and magnetic properties. It was found that these HEAs show high *M_s_*, low *B_r_*, and high *T_c_*, which indicate their potential application as soft magnetic materials. This paper offers a good method for designing future high-performance soft magnetic materials.

## 2. Materials and Methods

The FeCoNi(CuAl)_0.8_Sn*_x_* (0 ≤ *x* ≤ 0.10) HEAs were prepared via arc-melting the constituent elements of 99.99% purity using a water-cooled Cu crucible. The alloys were sucked into a 100 × 10× 2 mm water-cooled Cu mold after remelting four times. X-ray diffraction (XRD, D/max-2500 V, Rigaky Corporation, Tokyo, Japan) was used to characterized the crystal structures of the FeCoNi(CuAl)_0.8_Sn*_x_* (0 ≤ *x* ≤ 0.10) HEAs at a scan speed of 1°/min. Scanning electronic microscopy (SEM, Hitachis-3400N) was used to observe the morphology of the samples. Transmission electron microscope (TEM, JEM-2100F, JEOL, Ltd., Tokyo, Japan) was employed for the microstructure of HEAs. Dual-beam focused ion beam (FIB, FEI Helios 600i, Hillsboro, OR, USA) was used to prepare the TEM samples. The high angle annular dark field (HAADF) images were performed by a scanning transmission electron microscope with energy dispersive spectrometer (STEM/EDS, JEM-2100F, JEOL, Ltd., Tokyo, Japan). The *M_s_* and thermomagnetic curves was obtained from vibrating sample magnetometer (VSM, Lakeshore 7407, Westerville, OH, USA). The coercivity (*H_c_*), hysteresis losses (*P_u_*), remanence (*B_r_*), initial permeability (*μ_i_*), and maximum permeability (*μ_max_*) were obtained from hysteresis curves (DC) test system (HCTS, FE-2100SD, Forever elegance, Hunan, China) using a 27 × 9 × 1.7 mm rectangular sample at a magnetic field of 25 kA/m. The thermal stability was analyzed by differential scanning calorimeter (DSC, DIAMOND) at a heating rate of 10 K/min.

## 3. Results and Discussion

### 3.1. X-ray Diffraction

**Figure 1:**
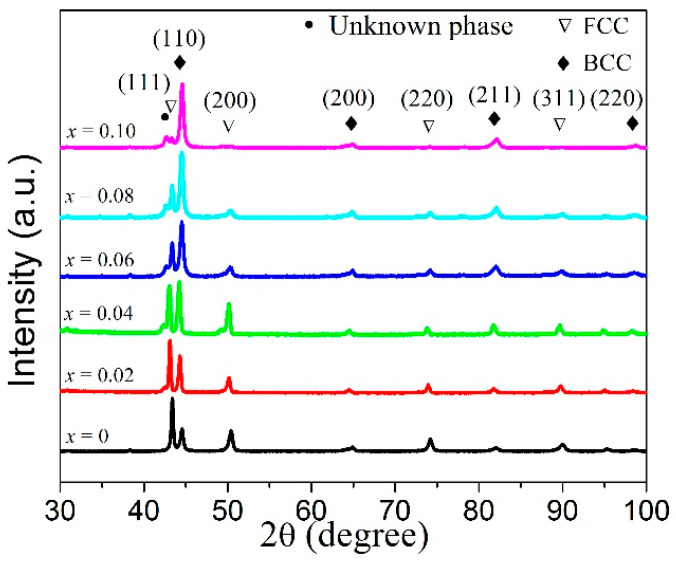
XRD patterns of FeCoNi(CuAl)_0.8_Sn*_x_* (0 ≤ *x* ≤ 0.10) high-entropy alloys (HEAs).

Figure 1 shows the XRD patterns of the FeCoNi(CuAl)_0.8_Sn*_x_* (0 ≤ *x* ≤ 0.10) HEAs. It is found that all of these HEAs consist of a mixed structure of FCC phase and BCC phase. A few diffraction peaks of unknown phases appear in the XRD patterns for x ≥ 0.02 and it is especially obvious for x ≥ 0.06. Here, I_(111)FCC_ and I_(110)BCC_ were used to denote the diffraction intensity of the strongest peak of (111) for FCC phase and (110) for BCC phase, respectively. Therefore, the relative content of FCC and BCC phases can be expressed as I_(110)BCC_/I_(111)FCC_. Table 1 shows the ratio of I_(110)BCC_/I_(111)FCC_. For x = 0, the ratio of I_(110)BCC_/I_(111)FCC_ is 0.38. This means the FCC phase in the FeCoNi(CuAl)_0.8_ HEA is a dominant phase. When the content of Sn increases from 0.02 to 0.10, the ratio of I_(110)BCC_/I_(111)FCC_ increases from 0.69 to 10.53, suggesting that the content of BCC phase increases rapidly. Based on XRD patterns, the lattice parameters of FCC and BCC phases can be calculated and shown in Table 1. It is seen that the lattice parameters of FCC and BCC phases all increase as *x* increases from 0 to 0.04, then they decrease and remain almost stable as *x* further increases. The decrease of lattice parameters of FCC and BCC phases may be due to the precipitation of these unknown phases.

### 3.2. Magnetic Properties

**Figure 2:**
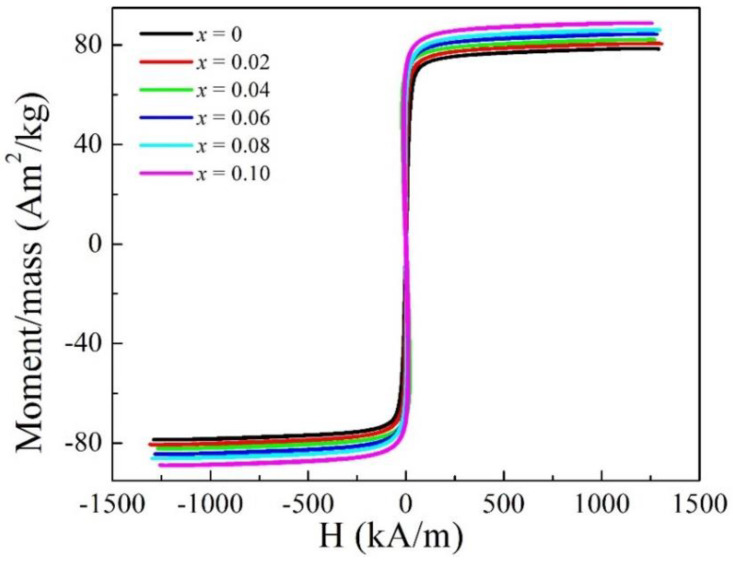
Hysteresis loops of FeCoNi(CuAl)_0.8_Sn*_x_* (0 ≤ *x* ≤ 0.10) HEAs measured by VSM.

**Figure 3:**
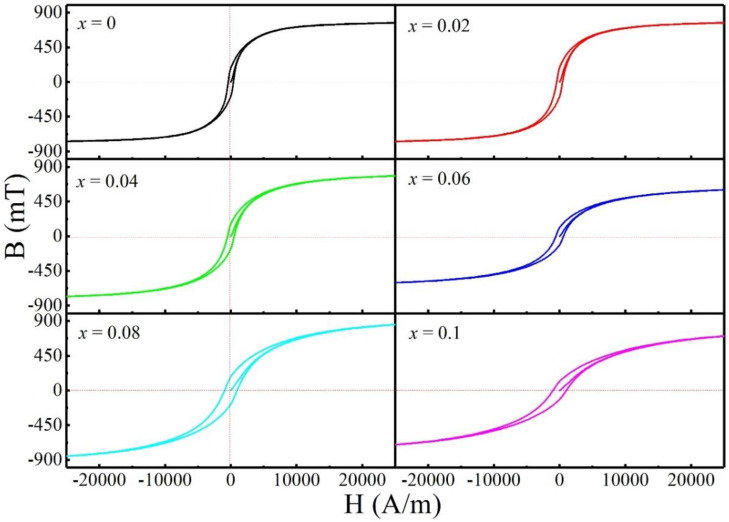
Magnetization curves and hysteresis loops of FeCoNi(CuAl)_0.8_Sn*_x_* (0 ≤ *x* ≤ 0.10) HEAs measured by HCTS.

**Figure 4:**
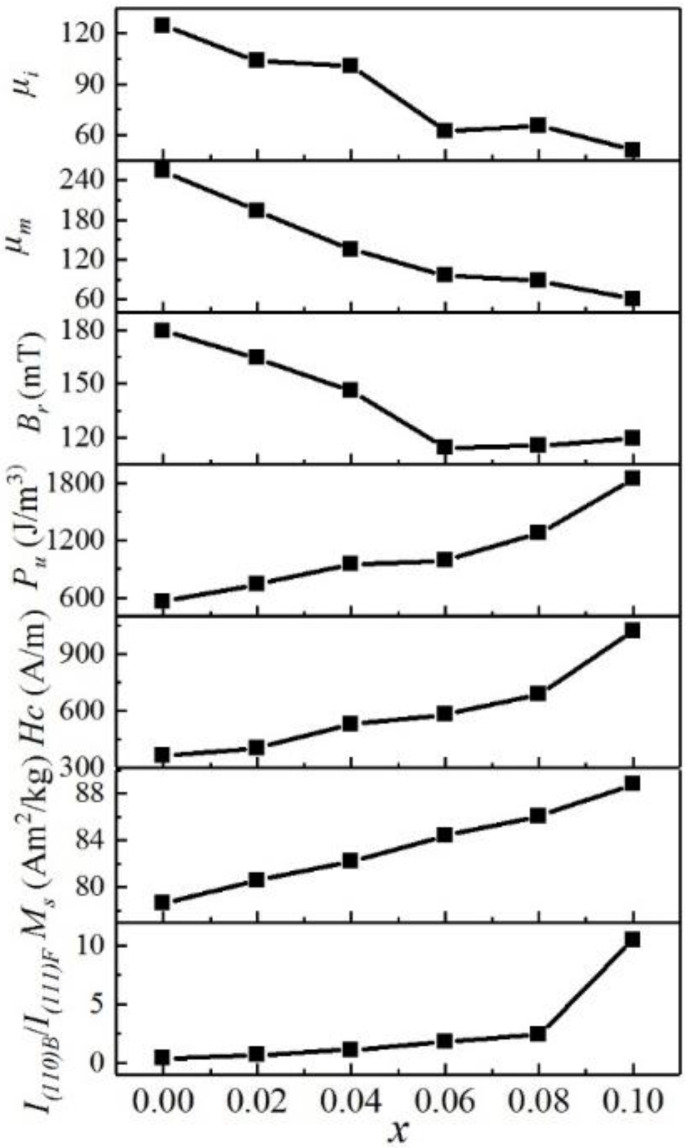
The ratio of I_(110)BCC_/I_(111)FCC_ and magnetic properties as a function of *x* for FeCoNi(CuAl)_0.8_Sn*_x_* (0 ≤ *x* ≤ 0.10) HEAs.

Figure 2 shows hysteresis loops of FeCoNi(CuAl)_0.8_Sn*_x_* (0 ≤ *x* ≤ 0.10) HEAs measured by VSM. Figure 3 shows the magnetization curves and hysteresis loops of FeCoNi(CuAl)_0.8_Sn*_x_* (0 ≤ *x* ≤ 0.10) HEAs measured by HCTS. The corresponding magnetic parameters obtained from Figure 2 and Figure 3 are shown in Table 2. As can be seen from Table 2, the value of *M_s_* increases from 78.6 Am^2^/kg to 88.8 Am^2^/kg as *x* increases from 0 to 0.10, which increases almost 13 percent. To make it easier to compare, the corresponding magnetic parameters as well as the ratio of I_(110)BCC_/I_(111)FCC_ as a function of *x* are shown in Figure 4. It is obvious that the *M_s_*, coercivity (*H_c_*), and hysteresis losses (*P_u_*) increase while the initial permeability (*μ_i_*) and maximum permeability (*μ_max_*) have a generally decreasing trend with increasing *x*. These results may be due to the increase of the volume fraction of BCC phase and the decrease of the volume fraction of FCC phase, which is in agreement with that of reported FeCoNi(CuAl)_0.8_Ga*_x_* (0 ≤ *x* ≤ 0.08) HEAs [25]. However, the value of remanence (*B_r_*) decreases after the addition of Sn into FeCoNi(CuAl)_0.8_ HEA, and this is completely different from the effect of Ga, which contributes to the increase of *B_r_* [25]. A careful analysis of these magnetic parameters shows the decrease of *B_r_* may be due to the rapid decrease of permeability.

**Figure 5:**
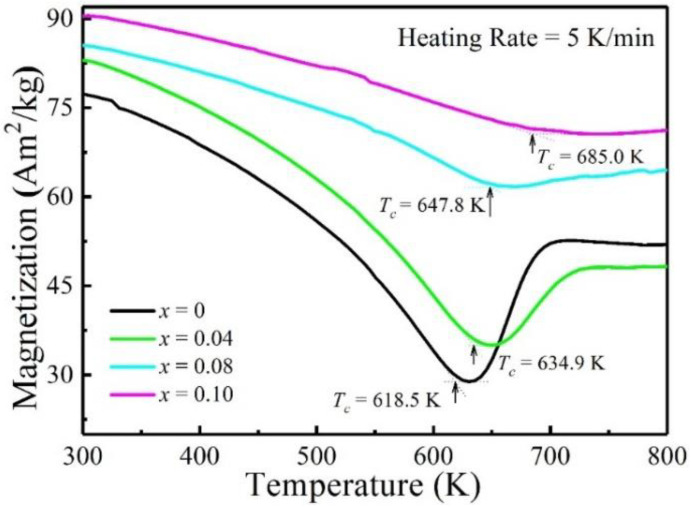
The thermomagnetic curves of FeCoNi(CuAl)_0.8_Sn*_x_* (0 ≤ *x* ≤ 0.10) HEAs.

The temperature dependence of magnetization for FeCoNi(CuAl)_0.8_Sn*_x_* (0 ≤ *x* ≤ 0.10) HEAs measured at an applied magnetic field of 1 T is shown in Figure 5. It can be seen that the magnetization of the FeCoNi(CuAl)_0.8_ HEA decreases first with increasing temperature, and it has hit bottom of 28.9 Am^2^/kg when the temperature is 630.4 K, then it increases quickly and reaches a constant value of about 52 Am^2^/kg at 706 K. It is worth noting that the magnetization does not reduce to 0 at its lowest point. That means a magnetic phase with higher Curie temperature exists in the alloy. As can be seen from Figure S1, there is one phase transformation peak [25] for FeCoNi(CuAl)_0.8_ HEA, at which the phase transforms from FCC to BCC phase. Therefore, it can be concluded that the Curie temperatures of BCC phase are obvious higher than that of FCC for FeCoNi(CuAl)_0.8_ HEA. That means the FCC phase exhibits paramagnetic behavior and a disordered magnetic structure while the BCC phase still shows ferromagnetic behavior with the increase of temperature. It also agrees well with our previous study [25]. Therefore, the magnetization of the alloy will increase when the temperature is above 630.4 K and below 706 K. The Curie temperature (*T_c_*), corresponding to the ferromagnetic to paramagnetic state transition of FCC phase for FeCoNi(CuAl)_0.8_ HEA, is indicated by arrow in Figure 5. For ease of comparison, the Curie temperatures of FCC phase for other FeCoNi(CuAl)_0.8_Sn*_x_* (0.02 ≤ *x* ≤ 0.10) HEAs are also shown in Figure 5. For *x* = 0.04, the outline of the curve is similar to that of the Sn-free alloy, but the Curie temperature of FCC phase increases significantly and reaches 634.9 K. For *x* ≥ 0.08, the Curie temperature of FCC phase continues to increase. However, there is only little change for the value of magnetization as the temperature continues to increase. This is because only a small number of FCC phase transform to BCC phase according to the results of XRD in Figure 1 and DSC curves in Figure S1. Therefore, it can be concluded that the addition of a minor amount of Sn can obviously increase the Curie temperature of FCC phase. The phases of the FeCoNi(CuAl)_0.8_Sn*_x_* (0 ≤ *x* ≤ 0.10) HEAs will transform from FCC with low *T_c_* to BCC phase with high *T_c_* at temperature of 600–700 K, which leads to the increase of magnetization. This provides a new idea for FeCoNi(CuAl)_0.8_Sn*_x_* (0 ≤ *x* ≤ 0.10) HEAs for their potential application as soft magnets to be used at high temperature.

### 3.3. Microstructure

**Figure 6:**
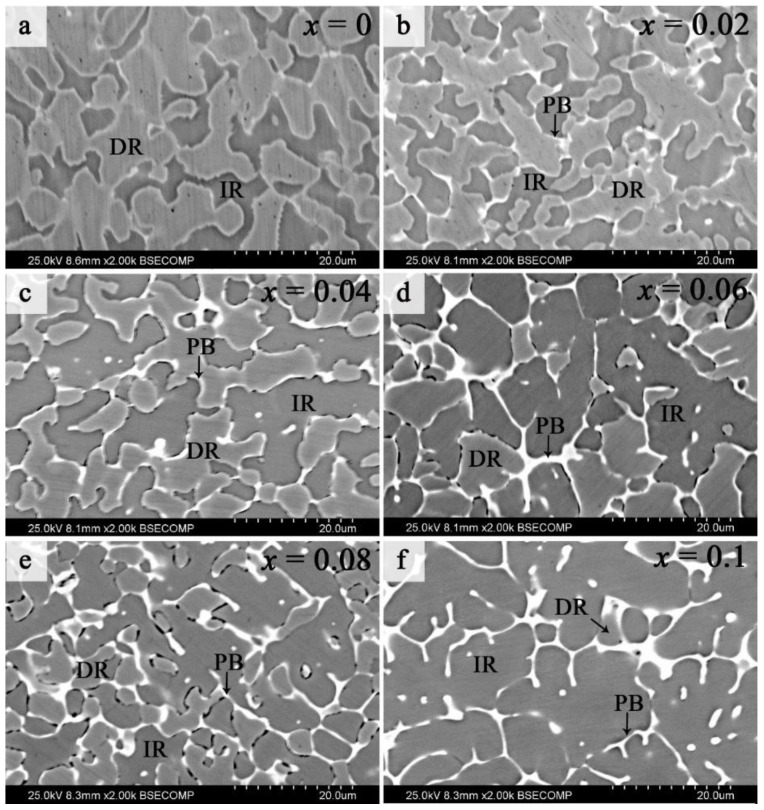
Typical SEM-BSE images of FeCoNi(CuAl)_0.8_Sn*_x_* (0 ≤ *x* ≤ 0.10) HEAs.

The SEM backscattered-electron (SEM-BSE) microstructures of the FeCoNi(CuAl)_0.8_Sn*_x_* (0 ≤ *x* ≤ 0.10) HEAs are displayed in Figure 6. In Figure 6a, two obviously identifiable contrasts are found in the Sn-free alloy, which can be identified as dendritic regions and interdendritic regions (marked as DR and IR, respectively). According to our previous studies [24,25], the DR and IR region can be confirmed to be FCC and BCC phase, respectively. For *x* ≥ 0.02, regions with strong contrast appear between the DR and IR regions, namely, the phase boundary regions, which can be marked as PB region. Moreover, the volume fraction of DR gradually decreases while the volume fraction of IR and PB increase with the increase of *x*. In addition, it is worth noting that the DR phases are almost invisible in the FeCoNi(CuAl)_0.8_Sn_0.1__0_ HEA (Figure 6f). The above results are in good agreement with the XRD results. Therefore, we can conclude that the addition of Sn in FeCoNi(CuAl)_0.8_Sn*_x_* (0 ≤ *x* ≤ 0.10) HEAs can promote the formation of BCC phases and PB phases.

**Figure 7:**
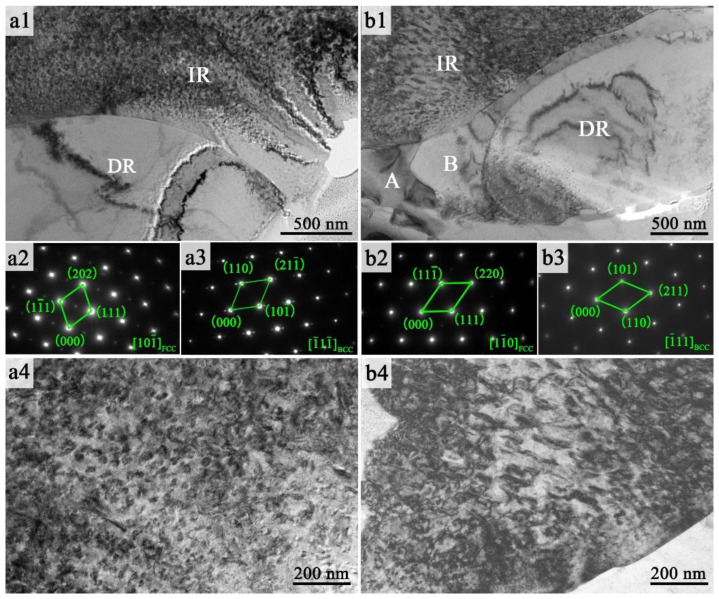
TEM images of FeCoNi(CuAl)_0.8_ HEA: (**a1**) bright-field image; (**a2**) SAED pattern of DR region; (**a3**) SAED pattern of IR region; (**a4**) high-magnification bright-field image of IR region. TEM images of FeCoNi(CuAl)_0.8_Sn_0.1__0_ HEA: (**b1**) bright-field image; (**b2**) SAED pattern of DR region; (**b3**) SAED pattern of IR region; (**b4**) high-magnification bright-field image of IR region.

In order to get more details of the FeCoNi(CuAl)_0.8_Sn*_x_* (0 ≤ *x* ≤ 0.10) HEAs, the structures of the FeCoNi(CuAl)_0.8_ and FeCoNi(CuAl)_0.8_Sn_0.1__0_ HEAs were further analyzed by TEM and the results are shown in Figure 7. Figure 7a1 confirms that FeCoNi(CuAl)_0.8_ HEA consists of two kinds of phases which are named as DR (dendritic region) and IR (interdendritic region) according to the results revealed by the typical SEM-BSM images (Figure 6). The selected-area-electron-diffraction (SAED) patterns of Figure 7a2,a3 suggest the FCC crystal structure of DR and the BCC crystal structure of IR in FeCoNi(CuAl)_0.8_ HEA. Meanwhile, it can be seen from Figure 7a1 that the surface of FCC phase is very smooth while the surface of BCC phase is much harsh. The high-magnification bright-field image of BCC phase for FeCoNi(CuAl)_0.8_ HEA is shown in Figure 7a4 and displays that the BCC phase contains a large number of nanoscale precipitates which distribute homogeneously in the BCC matrix. Moreover, the average size of nanoscale precipitates is 20 ± 5 nm. After the addition of Sn into the FeCoNi(CuAl)_0.8_ HEA, DR and IR regions can also be found in the FeCoNi(CuAl)_0.8_Sn_0.1__0_ HEA in Figure 7b1. Similarly, the former one can be indexed as FCC phase while the latter one is BCC phase according to the diffraction calibration in Figure 7b2 and Figure 7b3. In addition, it sees that two new regions (marked as A and B, respectively) are observed in Figure 7b1. We can infer that they are the source of the unknown phase peak in the XRD pattern. As shown in Figure 7b4, the high-magnification bright-field image of BCC phase for FeCoNi(CuAl)_0.8_Sn_0.1__0_ HEA is apparently different from that of the Sn-free alloy. The shape of the nano-precipitates is rod-like, and their density is lower than that in FeCoNi(CuAl)_0.8_ HEA. At the same time, the nanoprecipitates with an average length of about 100 nm and a width of about 30 nm are larger than that in FeCoNi(CuAl)_0.8_ HEA.

**Figure 8:**
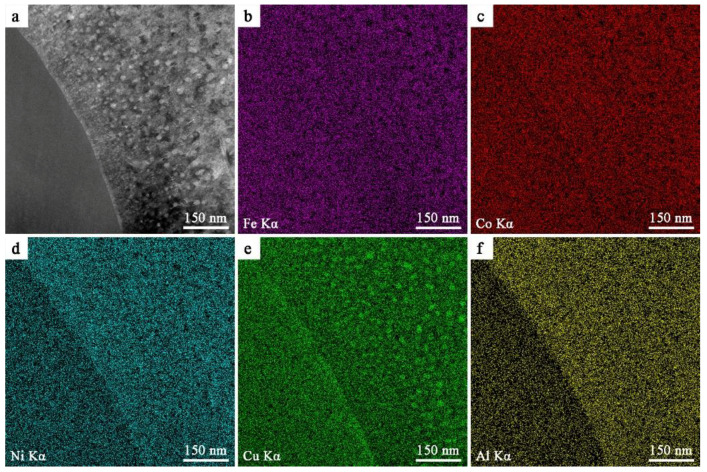
High angle annular dark field (HAADF) image (**a**) and elemental mapping images of FeCoNi(CuAl)_0.8_ HEA for Fe-Kα (**b**), Co-Kα (**c**), Ni-Kα (**d**) Cu-Kα (**e**) and Al-Kα (**f**).

The high angle annular dark field (HAADF) image and element mappings of Fe, Co, Ni, Cu, and Al for FeNiCo(CuAl)_0.8_ HEA measured by STEM-EDS technique are displayed in Figure 8. As can be seen from Figure 8a, the microstructure of the FeNiCo(CuAl)_0.8_ HEA is similar to that shown in Figure 7a1. It is worth noting that a phase boundary region with a width of about 13 nm can also be found in Figure 8a. We can confirm that these nanoprecipitates in the BCC region and the phase boundary region are rich in Cu (Figure 8e). Figure 8b–f suggests that the distribution of Fe and Co is very uniform in the alloy, while Ni and Al are enriched in the BCC region. The formation of the Cu-rich phase boundary regions in the as-cast FeNiCo(CuAl)_0.8_ HEA may be caused by the following three reasons. First, the melting point of Cu is lower than that of Fe, Co, and Ni [28], thus it may solidify after Fe, Co, and Ni when the temperature decreases. Second, the mixing enthalpies [28] (Table S1) between Cu and Fe, Co, and Ni are 13, 6, and 4 kJ/mol, respectively, meaning Cu is more likely to be repelled by other elements to form the Cu-rich phase boundary region. Finally, and most importantly, Cu and other elements are completely soluble at high temperature, but a large amount of Cu will precipitate out due to the rapid decrease of Cu solubility during casting. As for the formation of Cu-rich nano-precipitate in the BCC phase, it is due to the great difference in crystal structure between copper and the BCC phase matrix as well as the decrease of Cu solubility in BCC phase and the positive mixing enthalpies of Cu with Fe, Co, Ni, and Sn.

**Figure 9:**
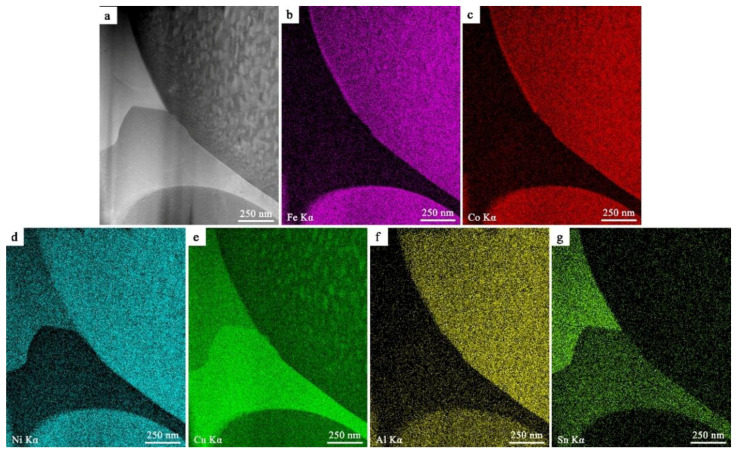
HAADF image (**a**) and elemental mapping images of FeCoNi(CuAl)_0.8_Sn_0.1__0_ HEA for Fe-Kα (**b**), Co-Kα (**c**), Ni-Kα (**d**) Cu-Kα (**e**), Al-Kα (**f**) and Sn-Kα (**g**).

Figure 9 shows the HAADF image and elemental mapping images of FeCoNi(CuAl)_0.8_Sn_0.1__0_ HEA. Compared with that of FeCoNi(CuAl)_0.8_ HEA, the microstructure shown in Figure 9a is more complex. From Figure 9a, a large number of Cu-rich nanoprecipitates with rod-like shape can be found in BCC phase. The shape of the precipitates changes to spherical as they approach the phase boundary region, and their size also gradually decreases. Figure 9b–g suggests that the distribution of Fe and Co is uniform in FCC and BCC phase regions, however, little Fe and Co can be found in the region between FCC and BCC phase. Moreover, the distribution of Ni and Al is enriched in BCC regions. One of the interesting things is that Cu and Sn segregate in the region between FCC and BCC phase. Besides, in the region where Sn is enriched, the content of Ni is also very high. Therefore, we can deduce that the unknown phase shown in the XRD patterns is composed of two phases, and one of it is rich in Cu, the other one is rich in Ni and Sn.

## 4. Conclusions

In this work, the FeCoNi(CuAl)_0.8_Sn*_x_* (0 ≤ *x* ≤ 0.10) HEAs were prepared by vacuum arc-melt casting. Effects of Sn content on the phase constitution and magnetic properties were studied. All the samples are composed of FCC and BCC phases, whereas some unknown phases appear with the addition of Sn. The addition of Sn promotes the formation of BCC phase, and it also affects the shape of Cu-rich nanoprecipitates in the BCC matrix. Moreover, the *M_s_* increases greatly while the remanence (*B_r_*) decreases with the increasing of *x* for FeCoNi(CuAl)_0.8_Sn*_x_* (0 ≤ *x* ≤ 0.10) HEAs. The addition of Sn can obviously increase the Curie temperature of the FCC phase. The phase of alloys with a mixture of FCC and BCC will transform from FCC to BCC phase at high temperature, leading to an increase of magnetization. They can be used as new soft magnetic materials at high temperatures.

## Figures and Tables

**Table 1:** The ratio of I_(110)BCC_/I_(111)FCC_ and lattice parameters of FeCoNi(CuAl)_0.8_Sn_*x*_ (0 ≤ *x* ≤ 0.10) HEAs.

*x*	I_(110)BCC_/I_(111)FCC_	a_FCC_ (nm)	a_BCC_ (nm)
0	0.38	0.3588	0.2856
0.02	0.69	0.3634	0.2891
0.04	1.11	0.3642	0.2894
0.06	1.81	0.3614	0.2876
0.08	2.40	0.3612	0.2879
0.10	10.53	0.3616	0.2876

**Table 2:** Magnetic parameters of FeCoNi(CuAl)_0.8_Sn*_x_* (0 ≤ *x* ≤ 0.10) HEAs.

*x*	*M_s_* (Am^2^/kg)	*B_r_* (mT)	*H_c_* (A/m)	*P_u_* (J/m^3^)	*μ_m_*	*μ_i_*
0	78.6	179.5	362.0	558.6	254.3	124.8
0.02	80.6	164.5	404.5	738.2	193.6	103.9
0.04	82.2	145.9	529.8	947.4	135.4	100.9
0.06	84.4	114.2	580.7	988.5	96.15	62.44
0.08	86.1	115.4	685.0	1275	87.69	65.55
0.10	88.8	119.5	1020	1848	60.86	50.94

## Supplementary Materials

The following are available online at http://www.mdpi.com/1099-4300/20/11/872/s1, Figure S1: DSC curves of FeCoNi(CuAl)_0.8_Sn*_x_* (0 ≤ *x* ≤ 0.10) HEAs. Table S1: The melting points (K) [28] of different elements and the mixing enthalpies (kJ/mol) [29] between two elements.
