# Supplementary material for: Magnetic Properties and Microstructure of FeCoNi(CuAl)0.8Snx (0 ≤ x ≤ 0.10) High-Entropy Alloys
Source: Entropy (Basel). 2018 Nov 13;20(11):872. doi: 10.3390/e20110872 (PMC7512446; doi:10.3390/e20110872)
Supplement: Supplementary file 1 [file entropy-20-00872-s001.pdf]

Supplementary Materials: Magnetic properties and microstructure of FeCoNi(CuAl)$_{0.8}$Sn$_x$ ($0 \le x \le 0.10$) High-Entropy Alloys

Zhong Li [1], Chenxu Wang [1], Linye Yu [1,2], Yong Gu [1,3], Minxiang Pan [1], Xiaohua Tan[1,*] and Hui Xu[1,*]

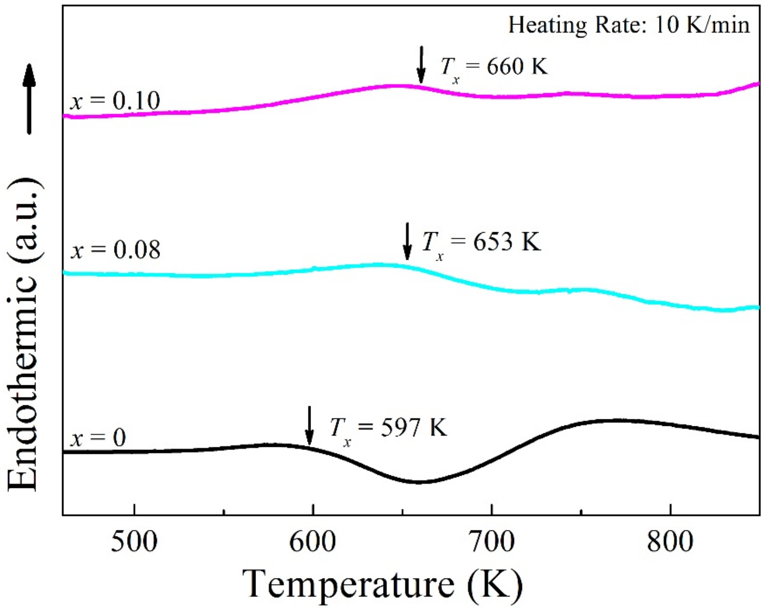

Figure S1. DSC curves of FeCoNi(CuAl)$_{0.8}$Sn$_x$ ($0 \le x \le 0.10$) HEAs

Table S1 The melting points (K) [28] of different elements and the mixing enthalpies (kJ/mol) [29] between two elements

Element	T_m/ K	Fe	Co	Ni	Cu	Al
Fe	1808	-	-	-	-	-
Co	1767	-1	-	-	-	-
Ni	1726	-2	0	-	-	-
Cu	1358	13	6	4	-	-
Al	933	-11	-19	-22	-1	-
Sn	505	11	0	-4	7	4
